# DNA Repair Gene (*XRCC1*) Polymorphism (Arg399Gln) Associated with Schizophrenia in South Indian Population: A Genotypic and Molecular Dynamics Study

**DOI:** 10.1371/journal.pone.0147348

**Published:** 2016-01-29

**Authors:** S. P. Sujitha, D. Thirumal Kumar, C. George Priya Doss, K. Aavula, R. Ramesh, S. Lakshmanan, S. Gunasekaran, G. Anilkumar

**Affiliations:** 1 Medical Biotechnology Division, School of Biosciences and Technology, VIT University, Vellore 632014, Tamil Nadu, India; 2 Department of Psychiatry, Government Vellore Medical College, Vellore, India; Sudbury Regional Hospital, CANADA

## Abstract

This paper depicts the first report from an Indian population on the association between the variant Arg399Gln of *XRCC1* locus in the DNA repair system and schizophrenia, the debilitating disease that affects 1% of the world population. Genotypic analysis of a total of 523 subjects (260 patients and 263 controls) revealed an overwhelming presence of Gln399Gln in the case subjects against the controls (P < 0.0068), indicating significant level of association of this nsSNP with schizophrenia; the Gln399 allele frequency was also perceptibly more in cases than in controls (p < 0.003; OR = 1.448). The results of the genotypic studies were further validated using pathogenicity and stability prediction analysis employing computational tools [I-Mutant Suite, iStable, PolyPhen2, SNAP, and PROVEAN], with a view toassess the magnitude of deleteriousness of the mutation. The pathogenicity analysis reveals that the nsSNP could be deleterious inasmuch as it could affect the functionality of the gene, and interfere with protein function. Molecular dynamics simulation of 60ns was performed using GROMACS to analyse structural change due to a mutation (Arg399Gln) that was never examined before. RMSD, RMSF, hydrogen bonds, radius of gyration and SASA analysis showedthe existence of asignificant difference between the native and the mutant protein. The present study gives astrong indication that the *XRCC1* locus deserves serious attention, as it could be a potential candidatecontributing to the etio-pathogenesis of the disease.

## Introduction

Schizophrenia is a debilitating neuropsychiatric disorder that affects approximately 1.0% of the population worldwide with devastating consequences for the affected individuals and their families [1]. Previous investigations addressing the causative factors of the disease reveal that the genes possessing Single Nucleotide Polymorphism (SNP) in the neurotransmitter system could become aberrant, and afford susceptibility to the disease [2, 3]. The SNPs of the serotoninergic system, for instance, have been shown to be associated with schizophrenia in various populations [4–6]. Lack of any disease-specific biomarker, however, is still a lingering problem that hinders proper diagnosis and treatment of the disease.

Previous investigators, in their quest to understand the etio-pathogenesis of the disease, have also been focusing on several putative targets other than the neurotransmitter and the neuroendocrine systems. The X-ray repair cross-complementation group (XRCC) of proteins, an essential component in the endogenous DNA repair system, encoded by the *XRCC* genes is now increasingly being considered a ‘potential target’ in this regard. The interest on the XRCC1 protein related to schizophrenia has been evolved out of its profuse presence (and the predominant mRNA levels) in the brains of rats and baboons [7–9]. Further, not only that the aberrations of the DNA repair system could restrain it from performing the repair function, but it would as well cause increased susceptibility to apoptosis, leading to the elimination of the damaged cell(s). Significantly, an increased rate of apoptosis has been observed in schizophrenia patients [10–13]. An interaction involving the SNP(s) of DNA repair system (*XRCC1*, for example) and increased apoptosis of nerve cells seems to exist, which in turn could play a vital role in the onset of several neuropsychological disorders, including schizophrenia. Further, there are also instances where polymorphism in the *XRCC1* locus is suggested to be entrained with neurological disorders [14–16]. This (hypothetical) relation offers a potential rationale to consider the *XRCC1* as a ‘candidate susceptibility gene’ for schizophrenia. Pertinently, the first report demonstrating an association between a variant at codon 399 of *XRCC1* locus and schizophrenia was brought out by Sadaat et al. (2008) [17] in Iranian population. However, no association was found to exist between Arg194Trp polymorphism of *XRCC1* and schizophrenia in the same population [18].

Earlier, Shen and colleagues [19] had identified three polymorphisms in the coding region of *XRCC1*, i.e., at codon 194 (Arg to Trp; UniProt ID: VAR_013400), at codon 280 (Arg to His; UniProt ID: VAR_013401), and at codon 399 (Arg to Gln; UniProt ID: VAR_011487). Studies have associated these three polymorphisms with breast cancer [20] and lung cancer [21,22].

To our knowledge, however, apart from the studies on Iranian population, attempts have not been made to explore the possible association of *XRCC1* with schizophrenia in any other population. It is at this juncture, that we present the results of our study on the frequency distribution of the SNP (Arg399Gln; rs25487) of *XRCC1* in Tamil population, and its association with schizophrenia.

Located on chromosome 19q13.2–13.3 and spanning 31.9 kb of genomic DNA, *XRCC1* is known to encode a protein of 633 amino acids, suggested to be involved in single-strand break repair, and base excision repair mechanisms [23]. The protein (XRCC1) has three functional domains, such as N-terminal domain (NTD) (1–151; 151 residues) that could interact with beta-polymerase (β-pol) [24], a central BRCT-I (breast cancer susceptibilityprotein-1) domain (315–403;88 residues), to interact with PARP [25], and a C-terminal BRCT-II (breast cancer susceptibilityprotein-2)domain (538–633; 96 residues), to interact with DNA ligase III [26], to facilitate the repair mechanism [27,28]. SNPs that occur in the conserved domain of *XRCC1* could result in the conformational change in the protein it encodes, resulting in deleterious effect, leading to genetic disease risk [29].

The present study will be the first report on the existence of a significant association between Arg399Gln of *XRCC1* and schizophrenia from Indian population. Also, we attempted to define the magnitude of deleteriousness of the variant protein through *in silico* prediction methods. Furthermore, molecular dynamics was initiated for the first time to reveal the impact of disease-contributing mutation Arg399Gln on the structure and function of the protein in question.

## Materials and Methods

### Sample collection

A total of 523 subjects (260 patients and 263 controls, with age ranging between 20 and 60) were recruited from among South Indian Tamil population for the present study with the following inclusion-exclusion criteria: The 260 case subjects, diagnosed with schizophrenia using Diagnostic and Statistical Manual for Mental Disorders 4^th^ edition (DSM-IV) criteria [30] by an experienced psychiatrist, were recruited at random from the Government Vellore Medical College. 263 Controls were chosen from healthy volunteers, based on the parameters (such as age, gender, sex and substance abuse), matching with those of the case subjects.

A questionnaire was designed to obtain the demographic data, including age, gender, marital status, mother tongue, status of current medication (for case subjects), age of onset of the symptoms (for case subjects), family history (if any) of any psychotic symptoms and smoking habits (if applicable) with regard to all the subjects (Table 1). While recruiting the subjects, a detailed enquiry was made for any possible existence of psychotic symptoms in the first degree and/or the second-degree relatives of all the subjects. It was ensured that those control subjects having the family history (in 1^st^ or 2^nd^ degree relatives) of any psychoticsymptoms were excluded from the study. Subjects who had any family history of carcinoma were also excluded from the study. While undertaking the present study, special care was taken to make sure that the subjects and the controls belong to only Tamil population. The homogeneity of the population was determined by the demographic data obtained from all the study subjects. It was ensured that the ancestors (for three generations) of the subjects, all of them living in villages in Tamil Nadu, were having Tamil as their mother tongue.

**Table 1:** Demographic details of the Patients and Controls.

Profile	Patients	Controls
Age	37.36±10.26 (Mean±SD)	32.19±11.13(Mean±SD)
Total Number of subjects	260	263
Male	170	171
Female	90	92
Married	219	227
Unmarried	41	36
Mother tongue	Tamil	Tamil
***Status of current medication***		
Risperidone	128	Not applicable
Clozapine	90	Not applicable
Chlorpromazine (CPZ)	10	Not applicable
Haloperidol	28	Not applicable
Risperidone+CPZ	4	Not applicable
***Age of onset of symptoms***		
Male	29.86± 9.39 (Mean±SD)	Not applicable
Female	30.68± 9.65 (Mean±SD)	Not applicable
***Pack-Years of smoked***	101(males)	90 (males)
<5 years	19	15
5–15 years	71	68
>15 years	11	7

10 ml of the peripheral blood was collected in Ethylene-diamine-tetra-acetic acid (EDTA)–coated vials from all the study subjects. All the subjects participated in the study voluntarily and signed the informed consent, the format of which was duly approved by the Institutional Review Boards of the respective institutions/hospitals, conforming to the Indian Council of Medical Research (ICMR) guidelines. Ethical Clearances were obtained from the respective institutions [Institutional Review Board (IRB), Government Vellore Medical College, Vellore, and University Human Ethical Committee, VIT University, Vellore] for collecting the blood samples, as per the guidelines stipulated by the Government of India.

### Genotyping

**Fig 1:**
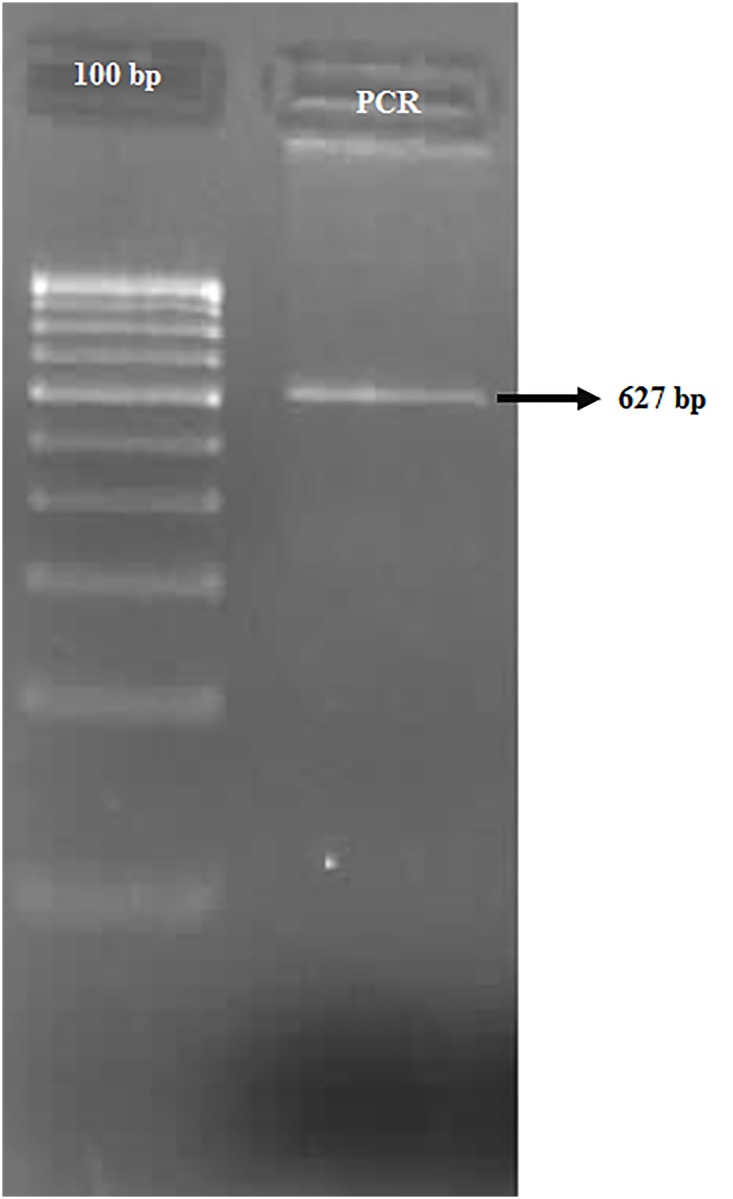
PCR amplicons visualized in 1.5% agarosegel. Lane 1- 100bp ladder; Lane 2- PCR product at 627 bp.

**Fig 2:**
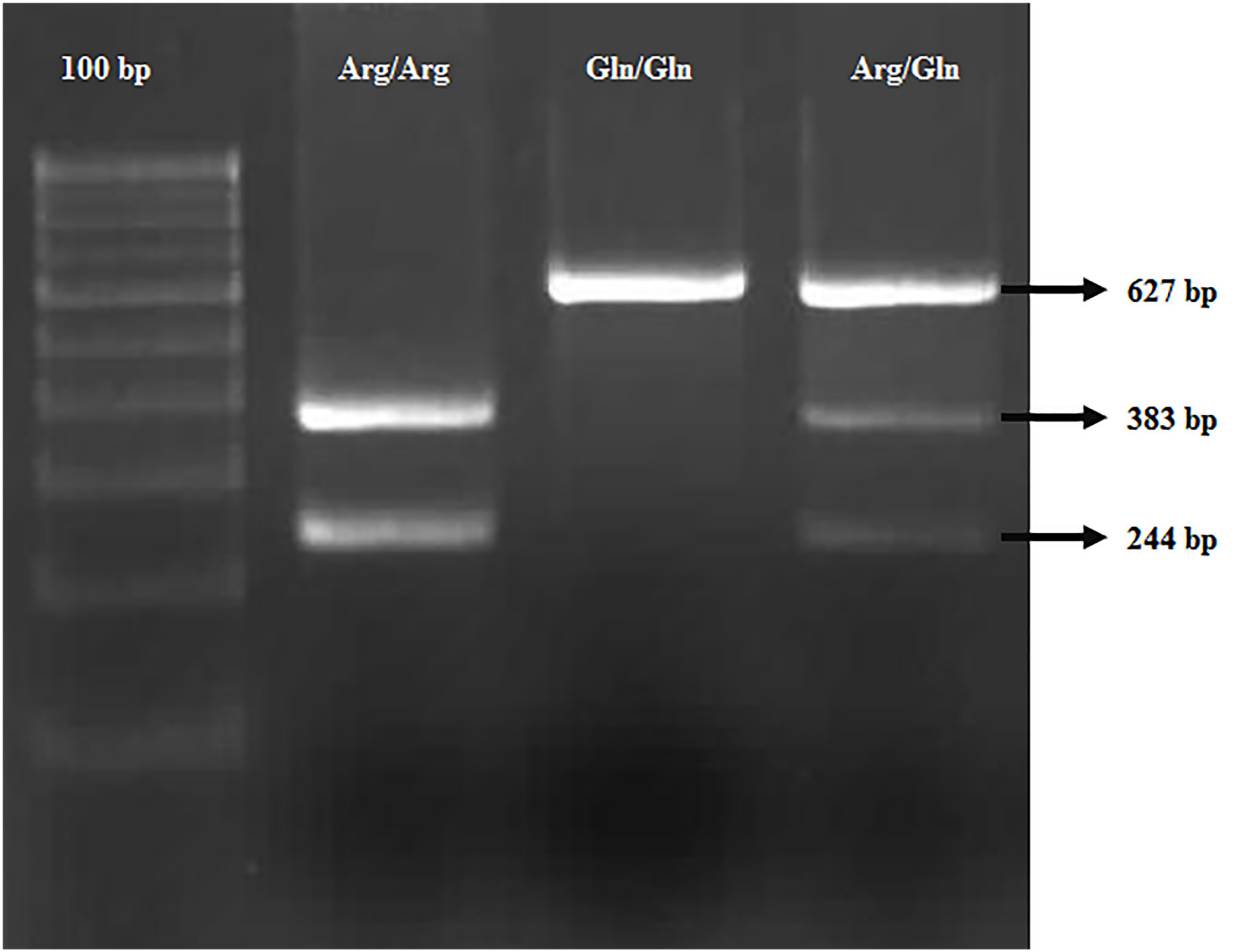
RFLP products visualized in 2.5% agarose gel. Lane 1- 100bp ladder; Lane 2- Arg/Arg genotype (wild); Lane 3- Gln/Gln genotype (mutant); Lane 3- Arg/Gln genotype (heterozygous).

Genomic DNA was isolated by phenol–chloroform method [31] from uncoagulated blood samples collected in EDTA–coated tubes. Genotyping (of Arg399Gln) was carried out by PCR amplification, followed by RFLP using *MspI* (NEB, England). The amplification reaction was performed in Applied Biosystems 9902 Thermal Cycler with a reaction volume of 15 μl containing 0.5 μl 100 pmol primer (IDT, USA, Fwd: 5’-GTTGTGCTTTCTCTGTGTCCA-3’; Rev: 5’-TCCTCCAGCCTTTTCTGATA-3’), 0.5μl 10mM dNTP mix (Merck), 0.5 μl 10x Taq buffer A (Merck), 0.5 μl Taq Polymerase (Merck) and 1μl template DNA (50 ng). The amplification protocol followed was: initial denaturation at 94°C (8 min), followed by 35 cycles of denaturation at 94°C (30 sec), annealing at 61°C (30 sec), initial extension at 72°C (30 sec), and final extension at 72°C (10 min). The amplified products were visualised in 1.5% agarose gel (Fig 1). The restriction digestion (*MspI*) was performed as per the manufacturer’s protocol, and the digested amplicon products (627 bp for Gln399Gln, 383 + 244 bp for Arg399Arg) were resolved on 2.8% agarose gel (Fig 2). The genotypic analysis performed with the Restriction Fragment Length Polymorphism (RFLP) method was followed by sequencing and proved to be consistent with that of the bands observed; the sequence information was deposited in NCBI GenBank, and the Accession Numbers were obtained.

### Statistical analysis

The data on allelic and genotypic frequencies were subjected to χ2 analysis (GraphPad Prism5.0) with a view toassess the differences (if any) in the proportionate distribution between the case subjects and the controls. That the genotype distribution would conform to the Hardy-Weinberg equilibrium was also assessed (http://ihg.gsf.de/cgi-bin/hw/hwa1.pl). The odds ratio and confidence limits were computed through GraphPad Prism 5.0. The power of the study was calculated by CaTs power calculator (http://www.sph.umich.edu/csg/abecasis/CaTS/). The sample size for the present study was estimated in such a way that the power of the study would be not less than 90%, for which the following parameters were considered: genetic relative risk = 1.5; prevalence of the disease = 0.01; risk allele frequency = value of the frequency observed in controls; α = 0.05 and multiplicative model of inheritance [32].

### *In silico*predictive mutational analysis

Five computational tools (I-Mutant Suite, iStable, PolyPhen2, SNAP, and PROVEAN) were employed for a predictive *in silico* analysis of the impact of themutation on the structure and function of the variant XRCC1 protein. Support vector machine (SVM) based tool, I-Mutant Suite (http://gpcr2.biocomp.unibo.it/cgi/predictors/I-Mutant3.0/I-Mutant3.0.cgi) predicts a variant based on protein stability. I-Mutant Suite which classifies the variants into three classes based on DDG value [such as neutral mutation (- 0.5< = DDG< = 0.5 kcal/mol), large decrease in stability (<-0.5 kcal/mol), and a large increase in stability (>0.5 kcal/mol)] [33] was also used for the present study. The free energy change (DDG) predicted by I-Mutant Suite is based on the difference between unfolding Gibbs free energy change of the mutant and that of the native protein (in kcal/mol). SVM–based iStable (http://predictor.nchu.edu.tw/iStable/indexSeq.php) integrates results from five different element predictors along with sequence information to predict the protein stability upon mutation in analmost accurate manner [34]. PolyPhen2 (http://genetics.bwh.harvard.edu/pph2/), through evolutionary sequence and structure-based approach, classifies a variant as ‘possibly damaging’, ‘probably damaging’ or ‘benign’, with the values ranging between 0 and 1; while the score range of 0.85–1 is being designated as ‘most likely damaging’, the score of 0.15–0.84 is designated as ‘possibly damaging’, and that with less than 0.14 is designated as ‘benign’ [35]. Neural network-based method screening for non–acceptable Polymorphism (SNAP) (https://www.rostlab.org/services/SNAP/) predicts the functional effect of a variant as ‘neutral’ or ‘non-neutral’, based on evolutionary sequence and structure information [36].

Assessing the single amino acid substitutions, small insertions and/or deletions, the Protein Variation Effect Analyzer (PROVEAN)(http://provean.jcvi.org/index.php) predicts the functional impact of protein sequence variations, as ‘deleterious’ or ‘neutral’ [37].

### *In silico*assessment of sequence conservation

Conservation pattern of a variant was calculated using an empirical Bayesian inference. Using the Consurf server (http://consurf.tau.ac.il/) that quantifies the degree of conservation at each aligned position; the extent of conservation of each residue is computed as a score range of 1–9; 1 denotes ‘rapidly evolving’ (variable) sites, 5 denotes sites that are ‘evolving at an average rate’, and 9 denotes ‘slowly evolving’ (evolutionarily conserved) sites [38].

### Structural visualization of mutant

Project “Have your Protein Explained” (HOPE) (http://www.cmbi.ru.nl/hope/home), an automatic mutant web server, was used to analyse the impact of the mutation on the protein structure. The web server utilizes FASTA, BLAST, UniProt, PDB (3D structure), YASARA (homology modelling), DSSP (secondary structure), HSSP (Conservation score), ClustalW (multiple sequence alignment), and DAS server (solvent accessibility) to make its prediction on protein structure [39].

### Structural analysis using molecular dynamics

For the structural analysis, the native (PDB ID: **2D8M)** was retrieved from PDB database (http://www.rcsb.org/pdb/home/home.do) followed by mutation [Arg399Gln) modelling with SWISS-PDB viewer [40]. For thecomputational investigation, MD simulation analysis was performed through Gromacs 4.5.6 package [41], on the native- and mutant type proteins, to observe if these mutations might lead to changes in the surface property and induce structural changes, to be propagated to distort the orientation of the protein functional site. The protein molecule was solvated in a rectangular box with water molecules (TIP3P) at 10 Å marginal radiuses. As the protein structures were found to be positively charged at physiological pH, the system was made electrically neutral, by the addition of 6 chlorine ions [Cl^-^] in the simulation box using the ‘genion’ tool, resulting in the replacement of water molecules by negative ions at the position of the first atoms with the most favourable electrostatic potential or at random position.Emtol convergence criterion was set to 1000 kcal/mol, and subsequently, the whole molecular system was subjected to energy minimization by steepest descent algorithm implementing GROMOS96 43a1 force field. Berendsen temperature coupling method [42] was used to regulate the temperature inside the box. Isotropic pressure coupling was performed using Parrinello-Rahman method [43]. Electrostatic interactions were computed using the Particle Mesh Ewald method [44]. The ionization states of the residues were set to appropriate to pH 7.0, with all histidine having been assumed neutral. The pressure was maintained at 1 atm with the allowed compressibility range of 4.5e-5 atm. SHAKE algorithm was used to constrain bond lengths involving hydrogen, permitting a time step of 2 fs. Van der Waals and Coulomb interactions were truncated at 1.0 nm. The non-bonded pair list was updated every 10 steps, and conformations were stored every 0.5 ps. The position restraint molecular dynamics simulation was performed for 20000 ps to soak the insolvent protein molecules to restrain the atoms at a fixed reference position. Finally, systems were subjected to MD simulation for 60 ns. The structural deviations in native and mutant XRCC1 structure were subjected to comparative analysis. g_rms compares the two structures by computing the root mean square deviation (RMSD) and the g_rmsf compute the root mean square fluctuation (RMSF). g_rms and g_rmsfgromacsbuilt-in tools were used for protein trajectories and atomic interaction analysis. Sumof hydrogen bonds formed by specific residues to other amino acids from the protein during the simulation were calculated by using g_hbond that analysed the hydrogen bonds between all possible donors and acceptors. g_sas was performed to determine the solvent accessible surface area. Finally,g_gyrate was used to study the protein compactness.

## Results

### Genotypic analysis

The genotypic and allelic frequency of rs25487 was assessed in all study cohorts. The frequency distributions of the genotypes were in accordance with Hardy-Weinberg Equilibrium (MAF>0.05; P>0.05) (in case subjects and the controls) (Tables 1 and 2). The genotypic analysis was confirmed by sequencing and proved to be consistent with that of the (restriction) digested PCR amplicons (Figs 1 and 2); the sequence information was deposited in NCBI GenBank, and the Accession Numbers were obtained [JX682562-TT /Gln haplotype (S1 File for sequence information)and KF848660-CC /Arg haplotype(S2 File for sequence information)]. The genotype frequency analysis revealed an overwhelming presence of Gln399Gln (36%) in the case subjects than in the control group (28%) (P<0.0068) (afterBonferroni correction), implicating an association of Gln399Gln with schizophrenia. Comparing the allelic frequencies between the cases and controls, the Gln399 allele (61%) frequency was evidently more in cases, than in controls (52%) (p<0.003; OR = 1.448, 95% CI = 1.132 to 1.851) (Table 2). The power analyses revealed that this study has a power of 90% to detect a genotype relative risk of 1.5 (α = 0.05) under a multiplicative model.

**Table 2:** Statistical evaluation on the genotype and allele frequency distribution.

***XRCC1* (rs25487)**	**Arg/Arg**	**Gln/Arg**	**Gln/Gln**	**χ2 & p value (df = 2)**	**P value of Hardy Weinberg Equilibrium**	**Arg**	**Gln**	**χ2 & p value (df = 1)**
**Controls**	62(0.23)	128(0.49)	73(0.28)	9.97 & 0.0068	0.71	252(0.48)	274(0.52)	8.74 & 0.003^a^ OR = 1.448;95% CI = 1.132 to 1.851
**Patients**	35(0.13)	132(0.51)	93(0.36)		0.29	202(0.39)	318(0.61)	
***XRCC1* (rs25487)**	**Arg/Arg**	**Gln/Arg**	**Gln/Gln**	**χ2 & p value (df = 2)**	**P value of Hardy Weinberg Equilibrium**	**Arg**	**Gln**	**χ2 & p value (df = 1)**
**Patients with Nicotine addiction**	15(0.15)	49(0.49)	37(0.37)	0.43 & 0.80	1.0	79(0.39)	123(0.61)	0.0096 & 0.92
**Patients without Nicotine addiction**	20(0.13)	83(0.52)	56(0.35)		0.24	123(0.39)	195(0.61)	
***XRCC1* (rs25487)**	**Arg/Arg**	**Gln/Arg**	**Gln/Gln**	**χ2 & p value (df = 2)**	**P value of Hardy Weinberg Equilibrium**	**Arg**	**Gln**	**χ2 & p value (df = 1)**
**Controls with Nicotine addiction**	22(0.24)	45(0.5)	23(0.26)	0.33 & 0.85	1.0	89(0.49)	91(0.51)	0.25 & 0.612
**Controls without Nicotine addiction**	40(0.23)	83(0.48)	50(0.29)		0.64	163(0.47)	183(0.53)	

P< 0.05 was considered as significant, C.I- Confidence Interval, OR- Odds ratio;

^a^—after Bonferroni correction

### Nicotine substance abuse and the variant genotype frequency

Encouraged by the observation of heavy smoking prevalence among schizophrenia patients [the rate of smoking among schizophrenia patients has been reported to be at least three to five times more than in the general population and addiction to smoking has been suggested to be an aetiological factor for the disease][45–48], the present study examines if there exists any association between addiction to smoking and the frequency distribution of Arg399Gln. Furthermore, the demographic data obtained from the case subjects in the present study also evinced the inclination of schizophrenia subjects towards nicotine abuse. However, our analysis of the data on the cigarette smoking did not reveal any significant association (χ^2^ = 1.20; P = 0.27) between the frequency of the variant (Arg399Gln) and cigarette smoking.

### *In silico* predictive functional analysis

Our stability and pathogenicity prediction analysis using the five tools (I-Mutant Suite, iStable, SNAP, PolyPhen2, and PROVEAN) revealed the mutation Arg399Gln to be more ‘deleterious’ and affecting the protein stability (Table 3). Structural stability analysis performed by I-Mutant Suite suggested a ‘large decrease’ in the stability of the mutant protein, borne out by the free energy change DDG value –2.23. The istable analysis also predicted a ‘decrease’ in the structure stability due to the mutant form. Furthermore, the functional analysis performed through PolyPhen2 classified the mutant (Arg399Gln) as “most likely damaging”, judged by the score of 0.979. SNAP analysis based on the evolutionary sequence and structure showed that the mutant (Arg399Gln) is ‘non- neutral’, suggesting that it could be deleterious. The PROVEAN analysis also predicted the mutation as deleterious.

**Table 3:** Deleterious effects prediction of rs25487 using *in silico* tools.

Prediction Tool	Interpretation
I-MUTANT 3	Decrease (-2.23)
I-STABLE	Decrease
PolyPhen 2	Most likely damaging (0.979)
SNAP	Non- neutral
PROVEAN	Deleterious

### Sequence conservation analysis

**Fig 3:**
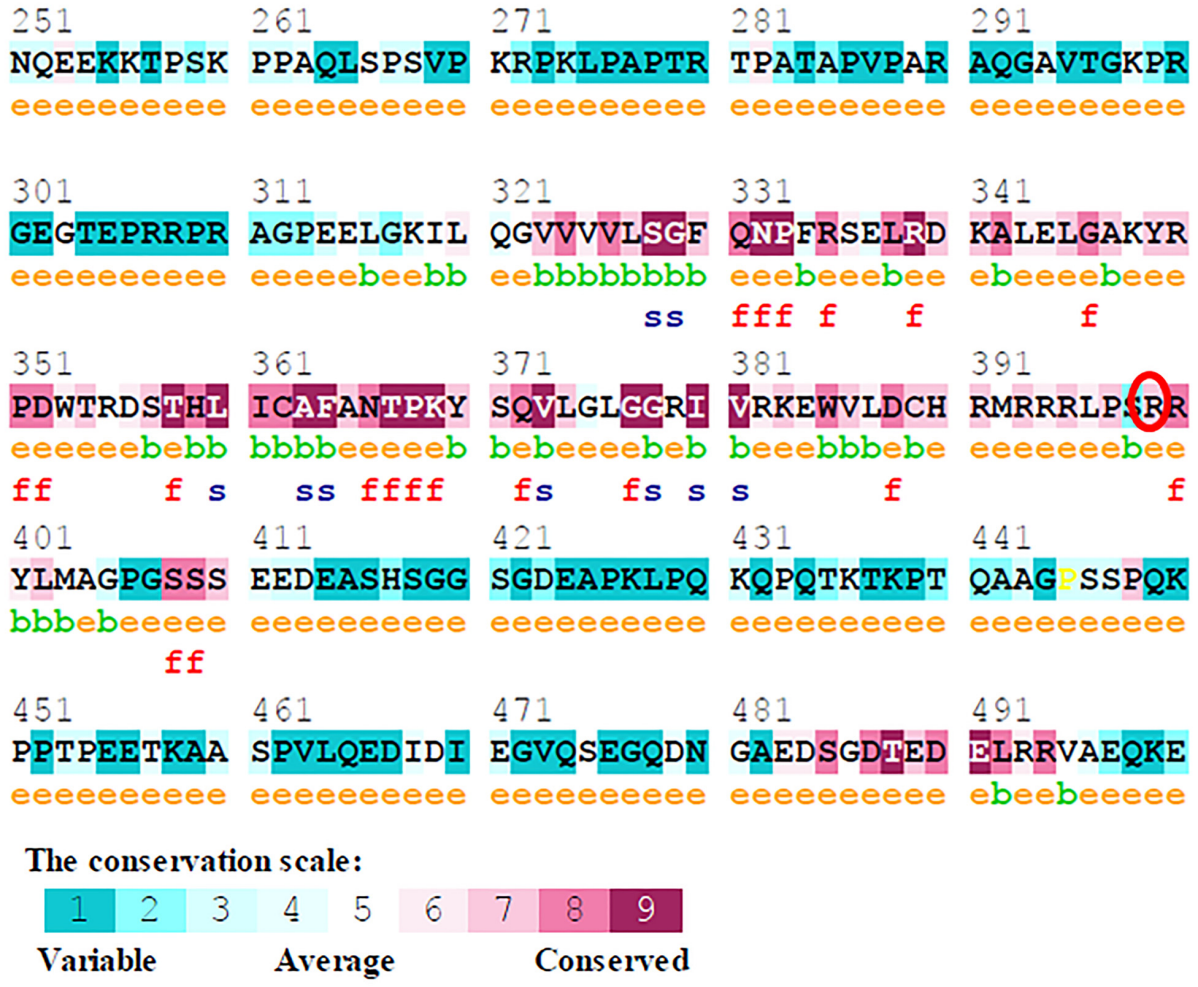
Sequence conservation result using Consurf tool, ‘R’ residue at 399 position indicates that the residue is conserved.

The conservation pattern of mutation at 399^th^ position in *XRCC1* was calculated using Bayesian classifier Consurf. This server provides the evolutionary conservation profile of amino acid by first identifying the conserved position using multiple sequence alignment and later estimates the evolutionary conservation rate using Empirical Bayesian. The analysis revealed a conservation score of 7.0, suggesting that the occurrence of mutation had taken place at a slowly evolving and evolutionarily conserved site (Fig 3).

### Structural visualization and insight using Molecular Dynamics

**Fig 4:**
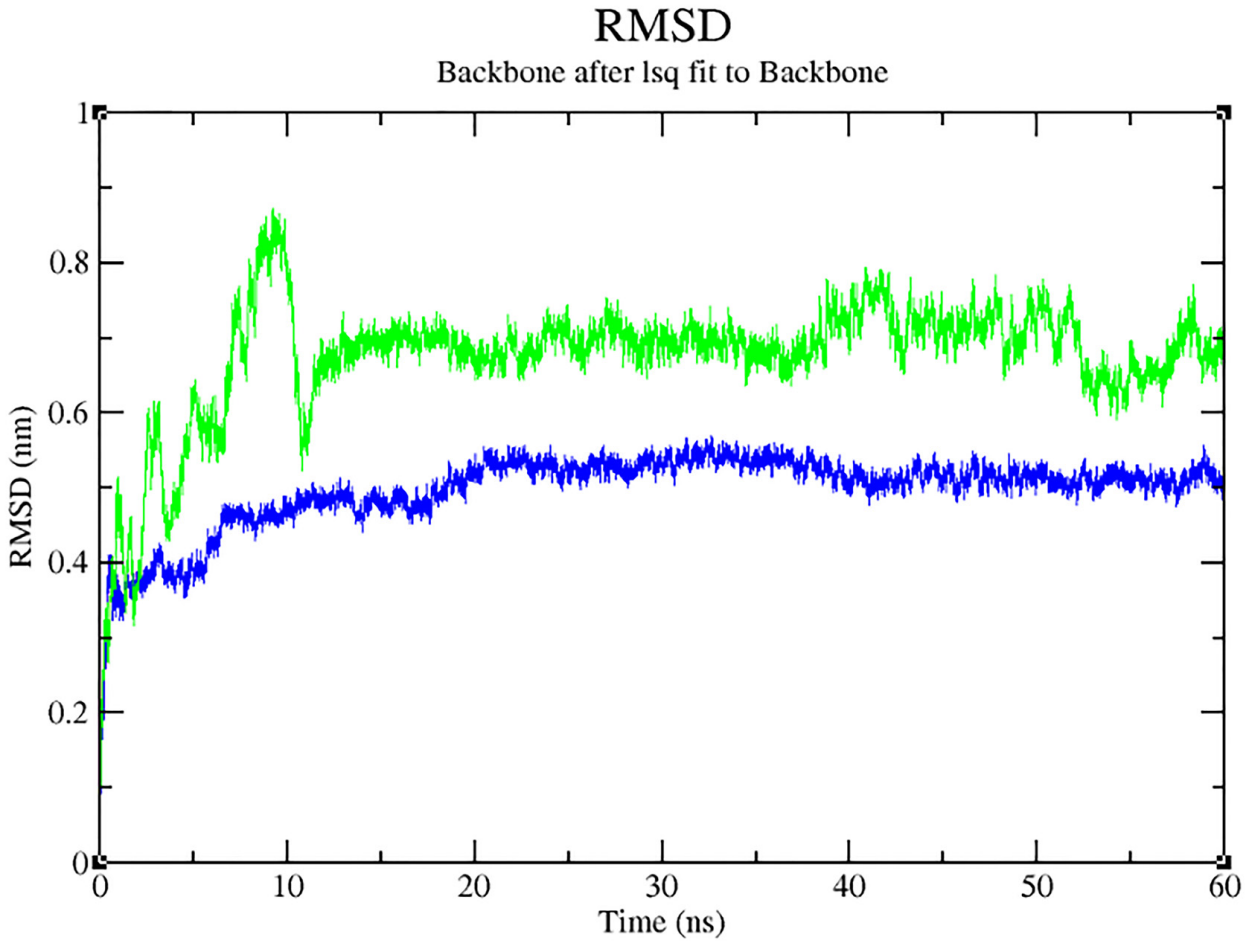
RMSD graph shows the deviation of the backbone in the native (blue) and the mutant (green) form of the XRCC1 protein during 60 ns simulation.

**Fig 5:**
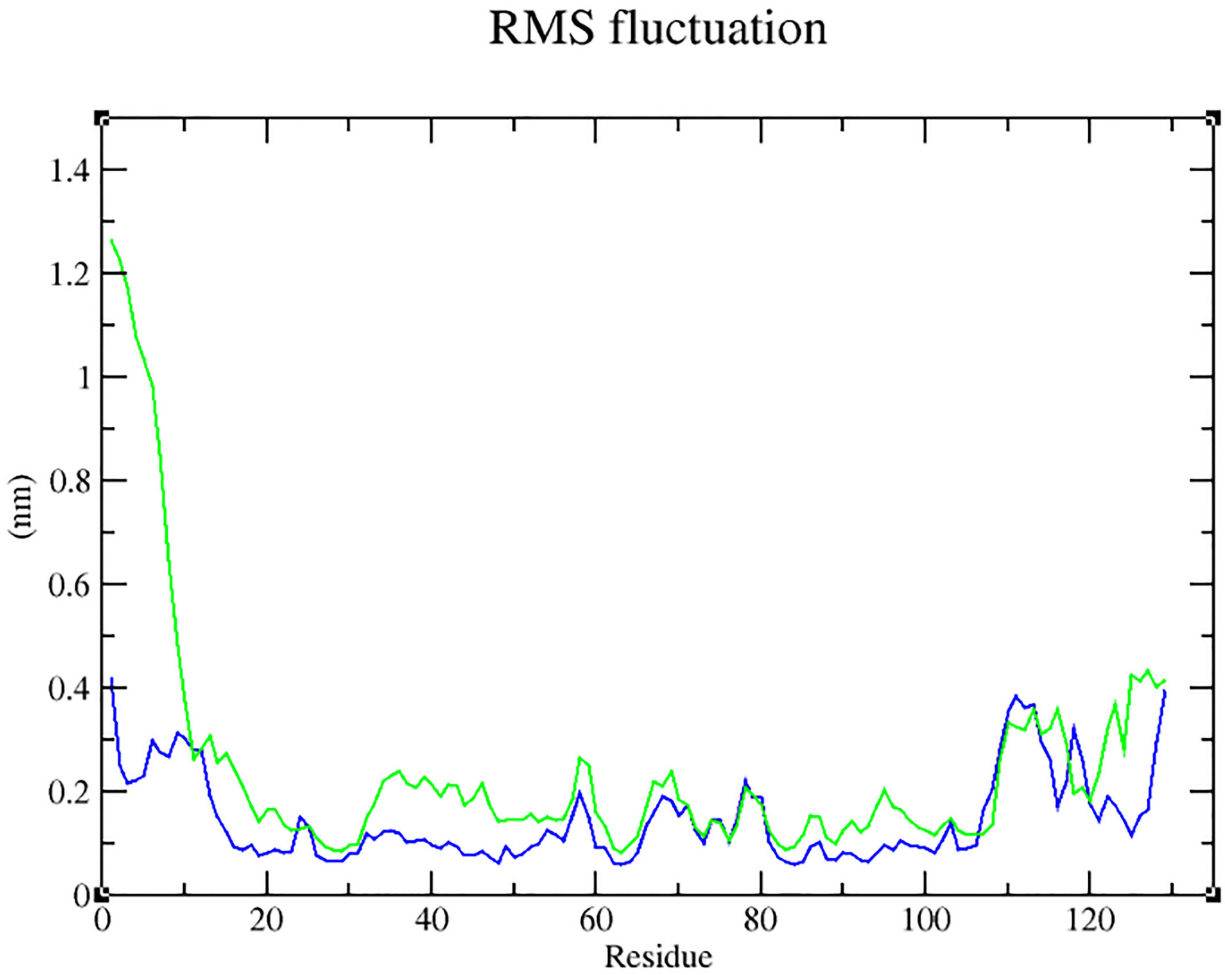
RMSF graph showing the carbon atom fluctuations in the native (blue) the mutant (green) forms of XRCC1 protein during 60 ns simulation.

**Fig 6:**
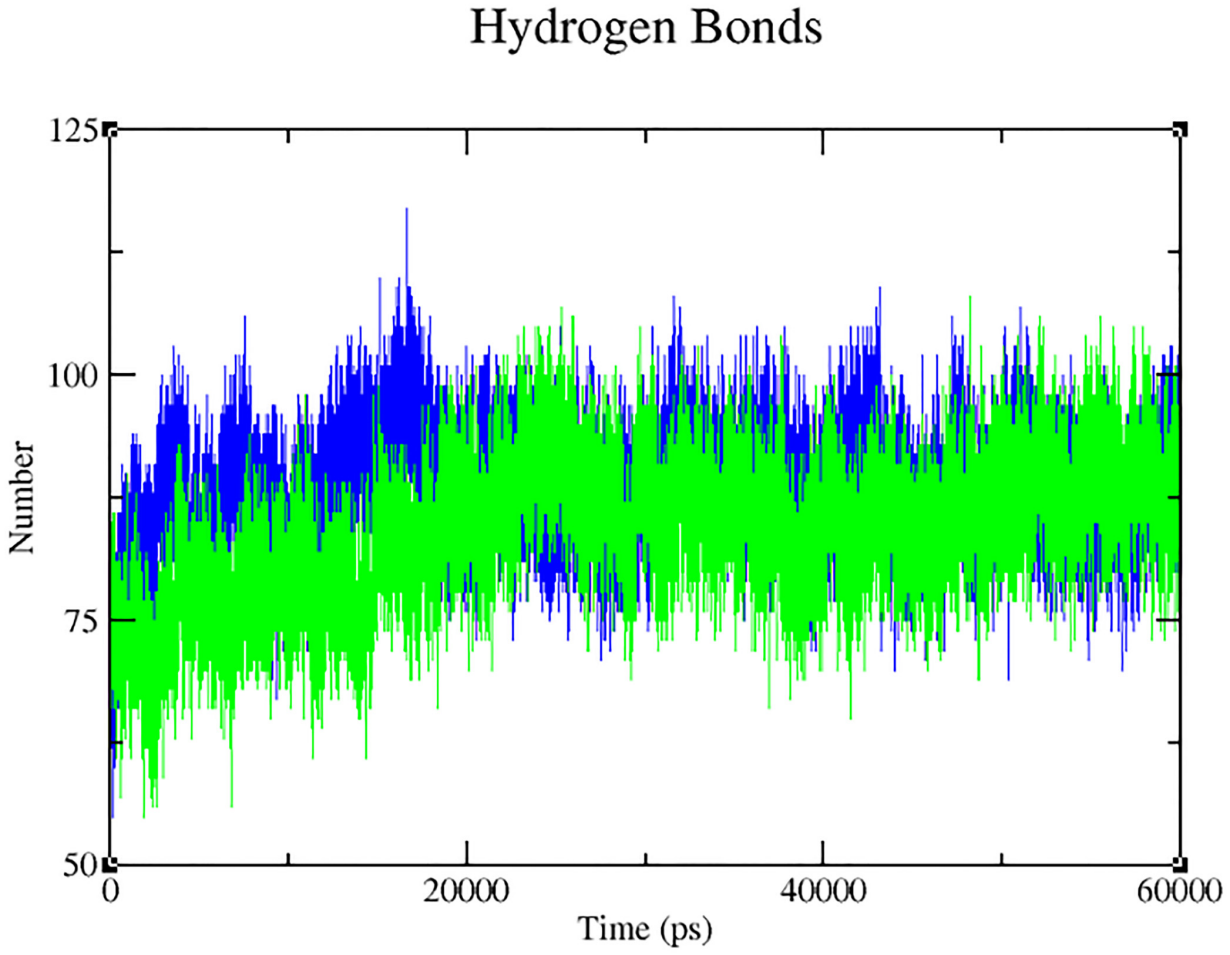
Hydrogen bond formation by the native (blue) and the mutant (green) form of XRCC1 during 60 ns simulation.

**Fig 7:**
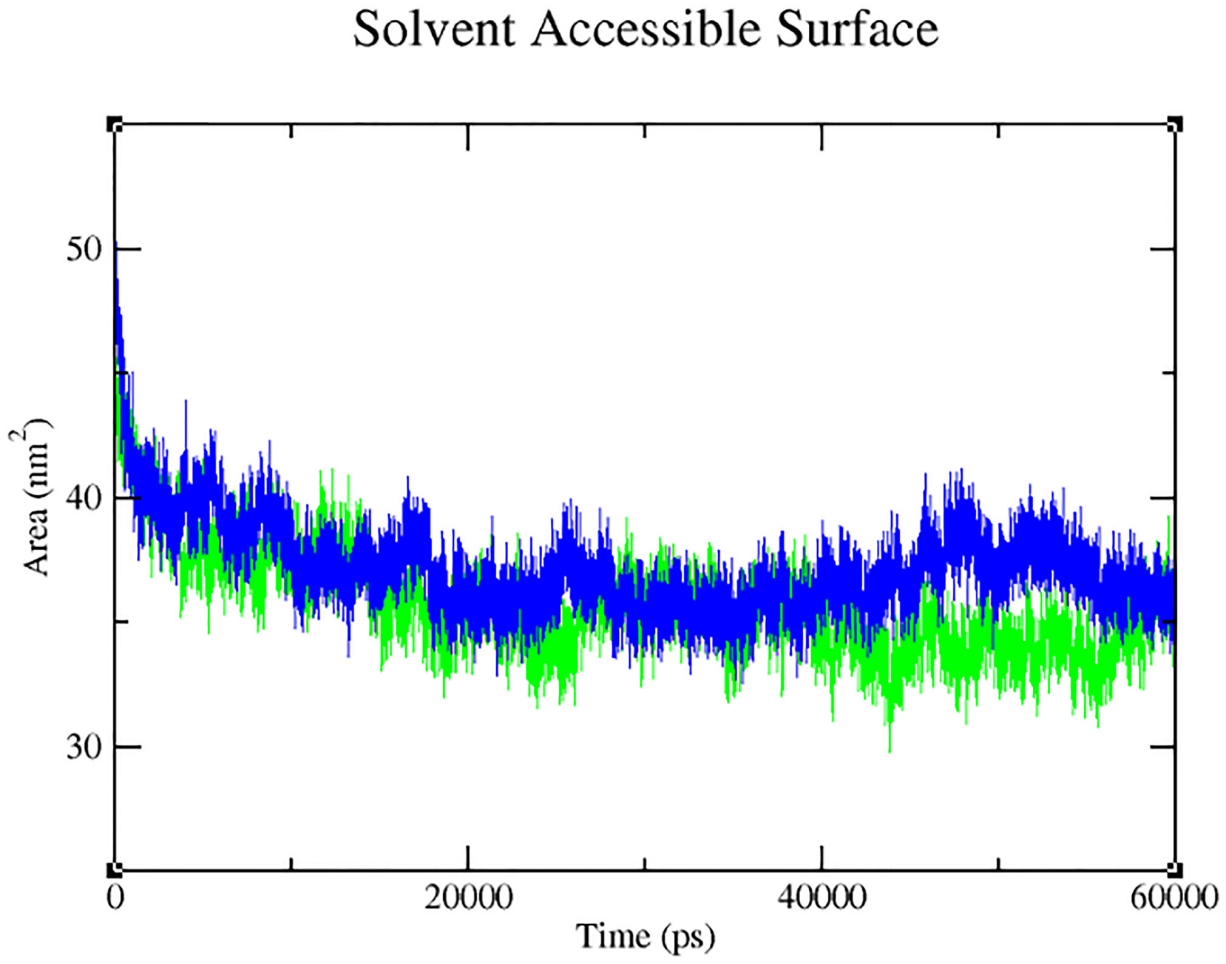
Solvent Accessible Surface formation by the native (blue) and the mutant (green) form of XRCC1 during 60 ns simulation.

**Fig 8:**
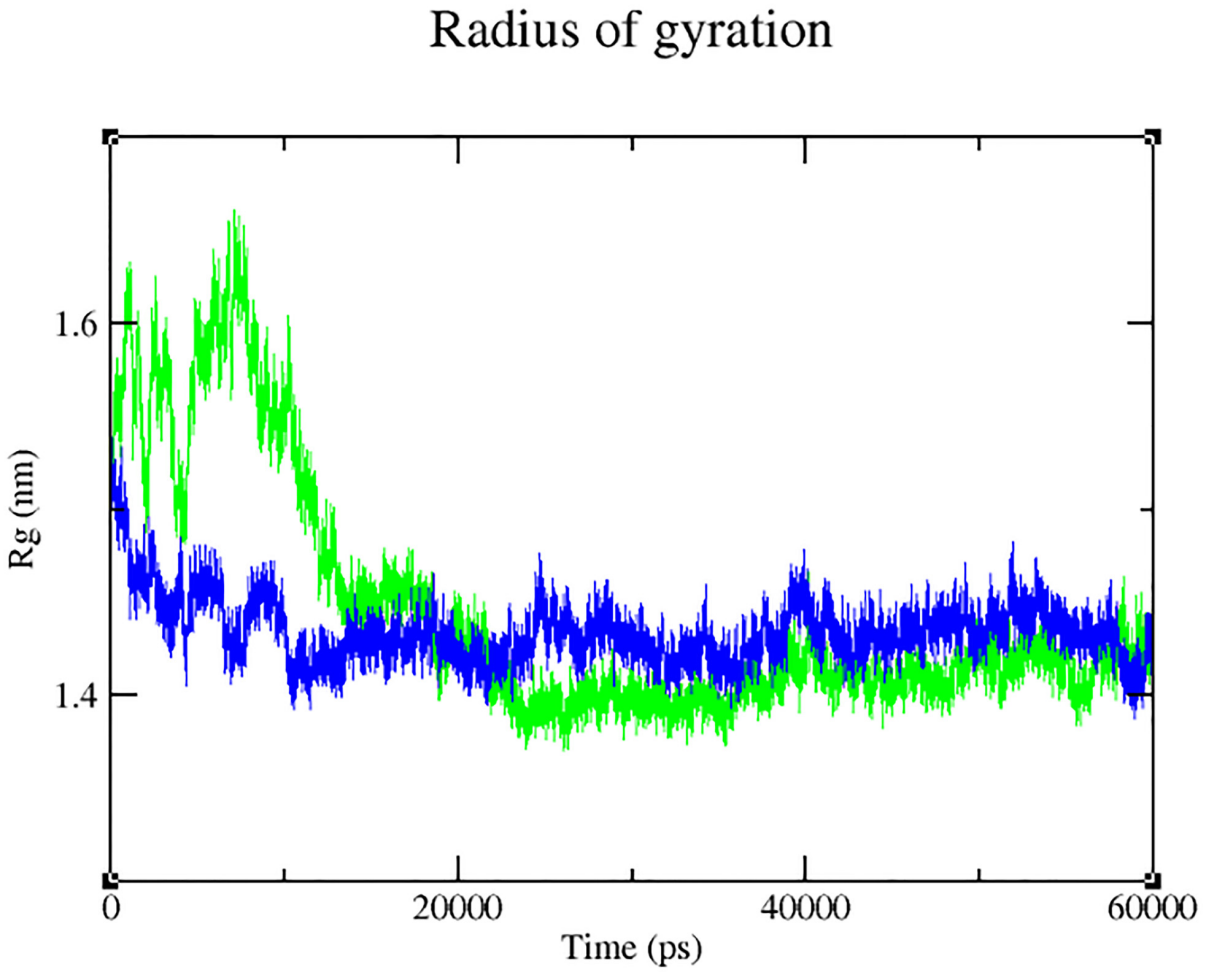
Radius of gyration by the native (blue) and the mutant (green) form of XRCC1 during 60 ns simulation.

Our analysis on the impact of mutation (through Project HOPE) revealed that the change in size, charge and hydrophobicity might lead to loss of interaction with other molecules or residues (Table 4), which in turn could result in undesirable effects. Taking into consideration of the parameters such as RMSD, RMSF, hydrogen bonds, Solvent- Accessible Surface Area (SASA) and radius of gyration, molecular dynamics simulation was performed for 60 ns with a view toexamine the change (if any) in conformation behaviour of the mutant protein Arg399Gln, as compared to that of the native. Both proteins experienced initial fluctuation due to the kinetic shock applied during the molecular dynamics simulation. Interestingly, the native and the mutant XRCC1 proteins showed distinctly different types of deviation throughout the entire simulation, resulting in backbone RMS fluctuation of ~0.15 nm. Overall, RMSD ranges of ~0.15–0.5 and ~0.12–0.82 nm were observed for the native and the mutant respectively (Fig 4). This magnitude of fluctuation, together with the difference between the average RMSD values after the relaxation period (20 ns), suggested that simulation produced stable trajectories, thus providing a suitable basis for further analyses. With the aim of determining whether mutation affected the dynamic behaviour of each residue, the RMSF values of Cα atom of native and mutant protein were calculated (Fig 5). The analysis revealed the existence of a higher degree of flexibility in mutant structure as compared to that of the native XRCC1 protein. Moreover, interestingly, the amino acid residues present in the region of 25 to 45 residues of the protein showed high fluctuation, indicating that the mutation has affected the protein conformation leading to an increased flexibility of residues in some regions while resulting in constraints on the flexibility of the residues in other regions. Results of the hydrogen bond analysis of the native and the mutant protein performed with respect to thetime indicated that the mutant Arg399Gln has significantly less number of hydrogen bonds formation during the entire simulation as compared to the native structure. The native protein has attained a maximum of ~120 hydrogen bonds while the minimum hydrogen bonds of the mutant have fallen behind ~60 (Fig 6). Solvent Accessible Surface of XRCC1 proteins in the native form showed relatively larger area (~35 to ~40 nm) and maintained the same throughout the simulation period. In the mutant form, however, the area was much less than ~35nm (Fig 7). Protein compactness was determined through the radius of gyration. The native R_g_ was found to be almost stable whereas the mutant has experienced continuous fluctuation all the way through; the fluctuation shown by the mutant could be due to the disturbance in compactness of the protein (Fig 8). This supports the results obtained through RMSD and RMSF.

**Table 4:** Structural visualization of the native and mutant protein forms using HOPE Server.

Amino acid properties	Native (Arg)	Mutant (Gln)	Mutation effect
**Size**	Large	Small	This might lead to loss of interactions
**Charge**	Positive	Neutral	This can cause loss of interactions with other molecules or residues
**Contacts**	More	Less	The wild type residue forms a salt bridge with the Glutamic acid on position 412. The difference in charge will disturb the ionic interaction made by the original, wild-type residue

## Discussion

This ‘population-based study’, depicts a first time report from an Indian population, on the existence of perceptibly high percentage of Gln399Gln genotypic (p<0.0068) and Gln399 allelic (61%; p<0.003) frequencies in case subjects than in controls, signifying the association between the SNP (rs25487) and schizophrenia. Although the SNPs at the serotoninergic and dopaminergic systems were suggested to have asignificant association with the disease [6, 49–52], not much was known of the association between the polymorphism(s) at *XRCC1* locus and schizophrenia. The maiden report from the Iranian population [17] wherein an association between aberrant *XRCC1* locus and schizophrenia has been suggested, and the present study on the Indian Tamil population strongly indicate that the gene(s) in the DNA repair mechanism (*XRCC1*) could be a potential candidate to be monitored in connection with the etio-physiology of this elusive disorder.

The question as to how mechanistically the aberration in *XRCC1* could contribute to the pathophysiology of schizophrenia is still enigmatic. Firstly, mRNA expression was found to occur predominantly in the brain, as evidenced from studies using baboon and rat models. Secondly, i*n vitro* and animal model studies revealed a preferential decrease in *XRCC1* mRNA in response to the use of DNA-damaging agents, reiterating its role in DNA repair[7,8]. Further, there is sufficient reason to believe that a damaged DNA repair system could promote apoptosis; thus the cell cycle progression is suggested to be arrested upon DNA damage, leading to apoptosis and elimination of the cell in question [53]. A correlation existing between polymorphism in *XRCC1Arg399Gln* and increased rate of apoptosisare reported in patients of ulcerative colitis[54]; and there are reports of increased susceptibility to apoptosis in schizophrenia patients [55, 56]. All these findings put together; it is tempting to hypothesize that a deficient DNA repair system (impaired *XRCC1*, for instance) could enhance apoptosis of the central nervous system (primarily the brain, evidenced by the reduced *XRCC* mRNA expression, as demonstrated in rats and baboons), leading to neuropsychological disorders, including schizophrenia.

It is worth noticing that the control subjects possessed 28% of the variant genotype (the susceptibility genotype for schizophrenia). However, despite this predisposition, this set of controls did not show any clinical symptom to the disease, the exact reason of which is not clear. Arguably, such carriers could have an increased susceptibility to the disease (than the rest of the controls), if exposed to the ‘pathogenic’ influence of the genetic and/or the epigenetic factors. A comparable situation has been reported in the Iranian population as well; the allelic frequency for the control population was 33% against the 40.8% in case subjects [17]. Pertinently enough, we are also encouraged to recall at this juncture, the instance of breast cancer studies in African Americans, wherein the variant 399 genotype showed significant association with breast cancer risk. Arg399Gln carriers, accounting for 26% of the controls (against 35% of the case subjects), however, did not clinically express the disease [57]. Here again, the authors suggest that such carriers could have an increased likelihood of developing the disease, apparently in combination with other alleles and/or environmental factors. In a complex genetic disease such as schizophrenia, the possible involvement of multiple factors in the etio-physiology of the disease cannot be ruled out.

This first ever *in silico* attempt to study the impact of Arg399Gln polymorphism on XRCC1 protein has provided us with valuable clues on the structural and functional discrepancies of the native and the mutant protein. The analysis has helped us understand the deleterious nature of the mutation (Table 3). A closer look at the mutant protein (Consurf Serverand Project HOPE) suggests that the differences in size, charge, hydrophobicity and surface property (compared to the native), could induce structural changes to distort the orientation of the protein functional site which in turn could render the mutant protein abnormal and deleterious. These observations, together with the results of the molecular dynamics studies implicate that the mutation (Arg399Gln) dramatically alters the structural and functional properties of the XRCC1 protein, and cause its malfunction and thus could contribute to the vulnerability to the disease.

Significantly, there has been a resurgence of interest in the recent past on the prevalence of cigarette smoking among schizophrenia patients [45, 58, 59]. Although there are suggestions to relate between genetic/environmental factors and nicotine addiction, and schizophrenia [45, 60](the exact reason for the heavy smoking dependence in schizophrenic patients, is still unclear. Not only that, cigarette smoking/nicotine exposure is suggested to alter the efficacy of the DNA repair gene [61], but meta-analysis studies [62,63] also revealed an association between the candidate polymorphism (Arg399Gln) and smoking behaviour. These interactions existing between schizophrenia and smoking behaviour, and the polymorphism in question have given us compelling reasons to examine if there exists any significant association between the aberrant *XRCC1* and smoking behaviour, leading to schizophrenia. Hence, the present study attempted to address the question whether the association of genetic predisposition (rs25487) along with nicotine addiction will increase the risk of schizophrenia or not. Further, some of the recent reviews even implicate that increased tobacco use could lead to the early manifestation of psychosis (including schizophrenia) [64,65]. However, despite the fact that the case subjects of our present study have shown anoverwhelming inclination towards nicotine abuse, our genotype analysis did not reveal any significant correlation between tobacco use (cigarette smoking) and the distribution of the variant genotype. This observation suggests that the mutation of the DNA repair system (Arg399Gln) could primarily be inherent in the patient (but not related to the nicotine substance abuse).

Another interesting aspect that merits discussion is the reduced cancer risk reported from among schizophrenia patients [66–68]. Although the precise mechanism underlying such a ‘cancer protection’ is unclear, it is apparent that the increased rates of apoptosis (one of the major lines of defence against cancer) prevalent among schizophrenia patients [56] may lead to the elimination of potential pre-malignant cells. At this juncture, a relatively recent experiment by Catts et al. [69] invites our attention, wherein the authors could observe significant differences in DNA damage response signallingin the unirradiated and irradiated lymphoblasts from the schizophrenia patients and the normal controls. In unirradiatedlymphoblasts, γH2AX levels (index of DNA double-stranded breaks) were significantly higher in the schizophrenia group, compared with those of the controls. In irradiated lymphoblasts, however, radiation-induced γH2AX levels were found to be significantly reduced in patients; resultantly, no difference was found to exist between the patients and the controls with respect to the rate of DNA repair or the cell cycle distribution. That the aberrant DNA damage response signalling could play any role in protecting the patients from cancer is still ambiguous, and would merit more research.

## Conclusion

To conclude, the association between the variant *XRCC1* and schizophrenia, as depicted in the present study, appears to be the rule at least in the context of (Indian) Tamil population, inasmuch as the mutant 399Gln is seen significantly prevalent in patients (evident from the genotype analysis), to impart deleterious influence (judged from the present *in silico* predictive mutational analysis). The present observation (on the deficient DNA repair system correlated with instances of schizophrenia) could attract further attention from researchers, as it could open up new candidate (gene), putatively pivotal to the etiology of the disease.

## Supporting Information

S1 File *XRCC1*- T allele, exon 10 partial sequence. (DOCX)

S2 File *XRCC1*- C allele, exon 10 partial sequence. (DOCX)

## References

[1] Ross CA, Margolis RL, Reading SA, Pletnikov M, Coyle JT. Neurobiology of schizophrenia. Neuron. 2006; 52:139–53. 17015232 10.1016/j.neuron.2006.09.015

[2] Molnar S, Mihanovi M, Grah M, Kezi P, Filakovi P, Degme D. Comparative study on gene tags of the neurotransmission system in schizophrenic and suicidal subjects. CollAnthropol. 2010; 34:1427–32. 21874733

[3] Chen J, Calhoun VD, Pearlson GD, Ehrlich S, Turner JA, Ho BC, et al The multifaceted genomic risk for brain function in schizophrenia. Neuroimage. 2012; 16: 866–75. 10.1016/j.neuroimage.2012.03.022 PMC3376184 22440650

[4] Contreras J, Hernández S, Quezada P, Dassori A, Walss-Bass C, Escamilla M, et al Association of serotonin transporter promoter gene polymorphism (5-HTTLPR) with depression in Costa Rican schizophrenic patients. J Neurogenet. 2011; 23: 83–9. 10.3109/01677060903583994 20397838

[5] Li W, Yang Y, Lin J, Wang S, Zhao J, Yang G, et al Association of Serotonin transporter gene (SLC6A4) polymorphisms with schizophrenia susceptibility and symptoms in Chinese-Han population. ProgNeuropsychopharmacolBiol Psychiatry. 2013; 44: 290–5. 10.1016/j.pnpbp.2013.04.003 23583772

[6] Sujitha SP, Nair A, Banerjee M, Lakshmanan S, Harshavaradhan S, Gunasekaran S, et al 5-Hydroxytryptamine (serotonin) 2A receptor gene polymorphism is associated with schizophrenia. Indian J Med Res. 2014; 140: 736–43. 25758572 PMC4365347

[7] Yoo H, Li L, Sacks PG, Thompson LH, Becker FF, Chan JY. Alterations in expression and structure of the DNA repair gene XRCC1. BiochemBiophys Res Commun. 1992; 186: 900–10. 10.1016/0006-291x(92)90831-5 1353960

[8] Zhou Z-Q, Walter CA. Expression of the DNA repair gene XRCC1 in baboon tissues. Mutat Res Lett. 1995;348: 111–6. 10.1016/0165-7992(95)00053-4 8524362

[9] Tebbs RS, Thompson LH, Cleaver JE. Rescue of Xrcc1 knockout mouse embryo lethality by transgene-complementation. DNA Repair (Amst). 2003;2: 1405–17. 14642568 10.1016/j.dnarep.2003.08.007

[10] Catts VS, Catts SV. Apoptosis and schizophrenia: is the tumour suppressor gene, p53, a candidate susceptibility gene? Schizophr Res. 2000;41: 405–15. 10728718 10.1016/s0920-9964(99)00077-8

[11] Catts VS, Catts SV, McGrath JJ, Féron F, McLean D, Coulson EJ, et al Apoptosis and schizophrenia: a pilot study based on dermal fibroblast cell lines. Schizophr Res. 2006;84: 20–8. 16626937 10.1016/j.schres.2006.03.016

[12] Jarskog LF, Glantz LA, Gilmore JH, Lieberman JA. Apoptotic mechanisms in the pathophysiology of schizophrenia. ProgNeuropsychopharmacolBiol Psychiatry. 2005;29: 846–58. 10.1016/j.pnpbp.2005.03.010 15908096

[13] Saadat M, Saadat S. Susceptibility To Breast Cancer And Intron 3 Ins/Del Genetic Polymorphism Of DNA Double-Strand Break Repair Gene XRCC4. J Med Biochem. 2015;34: 409–13. 28356849 10.2478/jomb-2014-0051 PMC4922352

[14] Qian Y, Chen W, Wu J, Tao T, Bi L, Xu W, et al Association of polymorphism of DNA repair gene XRCC1 with sporadic late-onset Alzheimer's disease and age of onset in elderly Han Chinese. J Neurol Sci. 2010; 15: 62–5. 10.1016/j.jns.2010.05.002 20553853

[15] Saadat I, Beyzaei Z, Aghaei F, Kamrani S, Saadat M. Association between polymorphisms in DNA repair genes (XRCC1 and XRCC7) and risk of preeclampsia. Arch. Gynecol Obstet. 2012; 286: 1459–62. 10.1007/s00404-012-2471-7 22825692

[16] Fu D, Calvo JA, Samso LD. Balancing repair and tolerance of DNA damage caused by alkylating agents. Nat Rev Cancer 2012; 12: 104–20. 10.1038/nrc3185 22237395 PMC3586545

[17] Saadat M, Pakyari N, Farrashbandi H. Genetic polymorphism in the DNA repair gene XRCC1 and susceptibility to schizophrenia. Psychiatr Res. 2008; 157:241–5. 10.1016/j.psychres.2007.07.014 17961713

[18] Derakhshandeh S, SaadatHassan I, Farrashbandi H, Saadat M. Association between genetic polymorphism of XRCC1 Arg194Trp and risk of schizophrenia. Psychiat Res. 2009; 169: 186. 10.1016/j.psychres.2009.03.022 19631990

[19] Shen MR, Jones LM, Mohrenweiser H. Nonconservative amino acid substitution variants exist at polymorphic frequency in DNA repair genes in healthy humans. Cancer Res. 1998; 58: 604–8. 9485007

[20] Jorgensen TJ, Helzlsouer KJ, Clipp SC, Bolton JH, Crum RM, Visvanathan K. DNA repair gene variants associated with benign breast disease in high cancer risk women. Cancer Epidemiol Biomarkers Prev. 2009; 18: 346–50. 10.1158/1055-9965.EPI-08-0659 19124519 PMC3428042

[21] Divine KK, Gilliland FD, Crowell RE, Stidley CA, Bocklage TJ, Cook DL, et al The XRCC1 399 glutamine allele is a risk factor for adenocarcinoma of the lung.MutatRes.2001; 5: 273–8. 10.1016/s0921-8777(00)00059-8 11104903

[22] Schneider J, Classen V, Helmig S. XRCC1 polymorphism and lung cancer risk. ExpertRev MolDiagn. 2008; 8:761–80. 10.1586/14737159.8.6.761 18999926

[23] Lamerdin J, Montgomery M, Stilwagen S, Scheidecker L, Tebbs R, Brookman K, et al Genomic sequence comparison of the human and mouse XRCC1 DNA repair gene regions. Genomics. 1995; 25: 547–54. 7789989 10.1016/0888-7543(95)80056-r

[24] Marintchev AMA, Mullen MW, Maciejewski B, Pan MR, Gryk Mullen GP. Site-directed mutagenesis analysis of the structural interaction of the single-strand-break repair protein, X-ray cross-complementing group 1, with DNA polymerase β. Nat Struct Biol. 1999; 6:884–93.10467102

[25] Masson MC, Niedergang V, Schreiber S, Muller J, Menissier-de Murcia, de Murcia G. XRCC1 is specifically associated with poly (ADP-ribose) polymerase and negatively regulates its activity following DNA damage. Mol Cell Biol. 1998; 18: 3563–71. 9584196 10.1128/mcb.18.6.3563 PMC108937

[26] Zhang X, Morera S, Bates PA. Structure of an XRCC1 BRCT domain: a new protein–protein interaction module. EMBO J. 1998; 17:6404–11. 9799248 10.1093/emboj/17.21.6404 PMC1170965

[27] Clements PM, Breslin C, Deeks ED, Byrd PJ, Ju L, Bieganowski P, et al The ataxia–oculomotor apraxia 1 gene product has a role distinct from ATM and interacts with the DNA strand break repair proteins XRCC1 and XRCC4. DNA. Repair. 2004; 3: 1493–1502. 15380105 10.1016/j.dnarep.2004.06.017

[28] Whitehouse CJ, Taylor RM, Thistlethwaite A. XRCC1 stimulates human polynucleotide kinase activity at damaged DNA termini and accelerates DNA single-strand break repair. Cell. 2001; 104:107–17. 11163244 10.1016/s0092-8674(01)00195-7

[29] Chacko P, Rajan B, Joseph T. Polymorphisms in DNA repair gene XRCC1 and increased genetic susceptibility to breast cancer. Breas Cancer Res Treat. 2005; 89: 15–21. 10.1007/s10549-004-1004-x 15666192

[30] American Psychiatric Association. Diagnostic and statistical manual of mental disorders (DSM-IV) 1994.

[31] Sambrook J, Russell DW. Molecular Cloning: A Laboratory Manual, 3rd ed New York: Cold Spring Harbor Laboratory 2001

[32] Skol AD, Scott LJ, Abecasis GR, Boehnke M. Joint analysis is more efficient than replication-based analysis for two-stage genome-wide association studies. Nat Genet. 2006; 38: 209–13. 16415888 10.1038/ng1706

[33] Capriotti E, Fariselli P, Rossi I, Casadio R. A three-state prediction of single point mutations on protein stability changes. BMC Bioinformatics. 2008; 9:S6. 10.1186/1471-2105-9-S2-S6 PMC2323669 18387208

[34] Chen CW, Lin J, Chu YW. iStable: Off-the-shelf Predictor Integration for Predicting Protein Stability Changes. BMC Bioinformatics. 2013; 14:S5. 10.1186/1471-2105-14-S2-S5 PMC3549852 23369171

[35] Adzhubei IA, Schmidt S, Peshkin L, Ramensky VE, Gerasimova A, Bork P, et al Functional annotations improve the predictive score of human disease-related mutations in proteins. Nat Methods. 2010; 7: 248–9.20354512

[36] Calabrese R, Capriotti E, Fariselli P Martelli PL, Casadio R. Functional annotations improve the predictive score of human disease-related mutations in proteins. Hum Mutat. 2009 30: 1237–44. 10.1002/humu.21047 19514061

[37] Choi Y, Sims GE, Murphy S, Miller JR, Chan AP. Predicting the Functional Effect of Amino Acid Substitutions and Indels. PLoS ONE. 2012; 7: e46688 10.1371/journal.pone.0046688 23056405 PMC3466303

[38] Landau M, Mayrose I, Rosenberg Y, Glaser F, Martz E, Pupko T et al ConSurf 2005: the projection of evolutionary conservation scores of residues on protein structures. Nucleic Acids Res. 2005; 33: W299–W302. 15980475 10.1093/nar/gki370 PMC1160131

[39] Venselaar H, TeBeek TA, Kuipers RK, Hekkelman ML, Vriend G. Protein structure analysis of mutations causing inheritable diseases. An e-Science approach with life scientist friendly interfaces. BMC Bioinformatics. 2010; 11:548 10.1186/1471-2105-11-548 21059217 PMC2992548

[40] Kaplan W, Littlejohn TG. Swiss-PDB Viewer (Deep View). Brief Bioinform. 2002; 2: 195–7. 10.1093/bib/2.2.195 11465736

[41] Hess B, Kutzner C, van der Spoel D E, Lindahl E. GROMACS 4: Algorithms for Highly Efficient, Load-Balanced, and Scalable Molecular Simulation. J Chem Theory Comput. 2008; 4: 435–47. 10.1021/ct700301q 26620784

[42] Berendsen HJC, Grigera JR, Straatsma TP. The missing term in effective pair potentials. J Phys Chem. 1987; 91: 6269–71.

[43] Parrinello M, Rahman. Polymorphic transitions in single crystals: A new molecular dynamics method. J Appl Phys. 1981; 52: 71–82.

[44] Essmann L, Perera ML, Berkowitz T, Darden H, Lee LG. Pedersen A smooth particle mesh Ewald method. J ChemPhy. 1995;103: 8577–93.

[45] Kelly C, McCreadie R. Cigarette smoking and schizophrenia. Adv. Psychiatr. Treat, 2000; 6: 327–31.

[46] de Leon J, Diaz F. A meta-analysis of worldwide studies demonstrates an association between schizophrenia and tobacco smoking behaviors. Schizophr Res. 2005;76:135–57. 15949648 10.1016/j.schres.2005.02.010

[47] Salin-Pascual RJ, Alcocer-Castillejos NV, Alejo-Galarza G. Nicotine dependence and psychiatric disorders. Rev Invest Clin. 2003; 55:677–93. 15011738

[48] Grant BF, Hasin DS, Chou SP, Stinson FS, Dawson DA. Nicotine dependence and psychiatric disorders in the United States: Results from the national epidemiologic survey on alcohol and related conditions. Arch Gen Psychiatry. 2004; 61:1107–15. 15520358 10.1001/archpsyc.61.11.1107

[49] Vijayan NN, Bhaskaran S, Koshy LV. Association of dopamine receptor polymorphisms withschizophrenia and antipsychotic response in a South Indian population. Behav Brain Funct. 2007; 3:34 17651483 10.1186/1744-9081-3-34 PMC1947997

[50] Neetha NV, Yoshimi, Linda VK, Chandrasekhar N, Chandrashekharan N. Evidence of association of serotonin transporter gene polymorphisms with schizophrenia in a South Indian population. J Hum Genet. 2009; 54:538–42. 10.1038/jhg.2009.76 19713975

[51] Melkersson K, Hulting AL. Serotonin receptor 2A gene polymorphisms and schizophrenia: association with family history, diagnostic subtype and height in patients. NeuroendocrinolLett. 2009; 30:343–51. 19855357

[52] Yao J, Pan Y, Ding M, Pang H, Wang B. Association between DRD2 (rs1799732 and rs1801028) and ANKK1 (rs1800497) polymorphisms and schizophrenia: A meta-analysis. Am J Med Genet B. 2015; 168:1–13. 10.1002/ajmg.b.32281 25504812

[53] El-Domyati MM, Al-Din A-BM, Barakat MT, El-Fakahany HM, Xu J, Sakkas D. Deoxyribonucleic acid repair and apoptosis in testicular germ cells of aging fertile men: the role of the poly(adenosine diphosphate-ribosyl)ation pathway. FertilSteril. 2009; 91: 2221–9. 10.1016/j.fertnstert.2008.03.027 18440520

[54] Bardia A, Tiwari SK, Gunisetty S, Anjum F, Nallari P, Habeeb MA, et al Functional polymorphisms in XRCC-1 and APE-1 contribute to increased apoptosis and risk of ulcerative colitis. Inflamm Res. 2012; 61: 359–65. 10.1007/s00011-011-0418-2 22193858

[55] Catts VS, Catts SV. Apoptosis and schizophrenia: is the tumour suppressor gene, p53, a candidate susceptibility gene? Schizophr Res. 2000;41: 405–15. 10728718 10.1016/s0920-9964(99)00077-8

[56] Catts VS, Catts SV, McGrath JJ, Féron F, McLean D, Coulson EJ, et al Apoptosis and schizophrenia: a pilot study based on dermal fibroblast cell lines. Schizophr Res. 2006;84: 20–8. 16626937 10.1016/j.schres.2006.03.016

[57] Duell EJ, Millikan RC, Pittman GS, Winkel S, Lunn RM, Tse CK, et al Polymorphisms in the DNA repair gene XRCC1 and breast cancer. Cancer Epidemiol Biomarkers Prev. 2001; 10: 217–22. 11303590

[58] Volkow ND. Substance use disorders in schizophrenia—clinical implications of comorbidity. Schizophr Bull. 2009; 35: 469–72. 10.1093/schbul/sbp016 19325163 PMC2669586

[59] Alderson HL, Lawrie SM. Does cigarette smoking cause psychosis? The lancet Psychiatry. 2015;2: 672–3. 10.1016/S2215-0366(15)00239-4 26249281

[60] Sagud M, Mihaljević-Peles A, Mück-Seler D, Pivac N, Vuksan-Cusa B, Brataljenović T, et al Smoking and schizophrenia. PsychiatrDanub. 2009; 21: 371–5. 19794359

[61] Hodgson ME, Poole C, Olshan AF, North KE, Zeng D, Millikan RC. Smoking and selected DNA repair gene polymorphisms in controls: systematic review and meta-analysis. Cancer Epidemiol Biomarkers Prev. 2010; 19: 3055–86. 10.1158/1055-9965.EPI-10-0877 20935063 PMC3108462

[62] Weng H, Weng Z, Lu Y, Nakayama K, Morimoto K. Effects of cigarette smoking, XRCC1 genetic polymorphisms, and age on basal DNA damage in human blood mononuclear cells. Mutat Res. 2009; 679: 59–64. 10.1016/j.mrgentox.2009.07.005 19628051

[63] Korytina GF, Akhmadishina LZ, Kochetova OV, Burdiuk IV, Aznabaeva IG, Zagidullin SZ, et al Association of the nicotine and cigarette smoke toxicants metabolic (CHRNA3/5, CYP2A6, NQO1) and DNA repair genes (XRCC1, XRCC3, XPC, XPA) with chronic obstructive pulmonary disease. MolBiol (Mosk). 48: 939–51. 25845234

[64] Myles N, Newall HD, Curtis J, Nielssen O, Shiers D, Large M. Tobacco use before, at, and after first-episode psychosis: a systematic meta-analysis. J Clin Psychiatry. 2012;73: 468–75. 10.4088/JCP.11r07222 22579146

[65] Gurillo P, Jauhar S, Murray RM, MacCabe JH. Does tobacco use cause psychosis? Systematic review and meta-analysis. The Lancet Psychiatry. 2015;2: 718–725. 10.1016/S2215-0366(15)00152-2 26249303 PMC4698800

[66] Cohen ME, Dembling B, Schorling JB. The association between schizophrenia and cancer: a population-based mortality study. Schizophr Res. 2002;57: 139–46. 12223244 10.1016/s0920-9964(01)00308-5

[67] Grinshpoon A, Barchana M, Ponizovsky A, Lipshitz I, Nahon D, Tal O, et al Cancer in schizophrenia: is the risk higher or lower? Schizophr Res. 2005;73: 333–41. 15653279 10.1016/j.schres.2004.06.016

[68] Catts VS, Catts S V, O’Toole BI, Frost ADJ. Cancer incidence in patients with schizophrenia and their first-degree relatives—a meta-analysis. ActaPsychiatr Scand. 2008;117: 323–36. 10.1111/j.1600-0447.2008.01163.x 18331573

[69] Catts VS, Catts SV, Jablensky A, Chandler D, Weickert CS, Lavin MF. Evidence of aberrant DNA damage response signalling but normal rates of DNA repair in dividing lymphoblasts from patients with schizophrenia. World J Biol Psychiatry. 2012;13: 114–25. 10.3109/15622975.2011.565073 21830993

