# Supplementary material for: DNA Repair Gene (XRCC1) Polymorphism (Arg399Gln) Associated with Schizophrenia in South Indian Population: A Genotypic and Molecular Dynamics Study
Source: PLoS One. 2016 Jan 29;11(1):e0147348. doi: 10.1371/journal.pone.0147348 (PMC4732819; doi:10.1371/journal.pone.0147348)
Supplement: S2 File — (DOCX) [file pone.0147348.s002.docx]

**S2 File: *XRCC1*- C allele, exon 10 partial sequence**

TTTCTCCCAGGCCTTTTCTGATAAGCAGGCTTCACAGAGCCCTCTGTGACCTCCCAGGCAGGTCCTCCTT

CCCTCATCTGGAGTACCCCAGCCCCTGCCCCGCTCCTCTCAGTAGTCTGCTGGCTACTCGGGCTGGGACC

ACCTGTGTTCTCCCGCTGGCAGGCCCCAGTCTGACTCCCCTCCAGATTCCTGGCATTGCCCAGCACAGGA

TAAGGAGCAGGGTTGGCGTGTGAGGCCTTACCTCCGGGAGGGCAGCCGCCGACGCAGCGGTGACAGTCCA

GCACCCACTCCTTACGCACGATGCGGCCTCCCAGGCCTAGGACCTGGCTGTACTTGGGGGTGTTGGCAAA

GGCACAGCTGGTGGGGGGCAGAAGTGAAGATGCCAGTTAGGTGTGATCTGAGGGGCAAAGGGGAATGAGG

ACAGGGGAGACAGACAGAGAGAGAGAGAGACAGACAGACAGACAGATCATGAGATTGAGGTGGGAGAAAC

AAAAAGAGGTTGGAGACCAAGGGAGAGATGCAAAAATCAGAGAGAAGACAAAGGCTACAGAATGCAGTGA

GAAAGAAAGCAAGTGAGAGAGAGACAAACCACCTGCTAGCATAGTGGACACAGAGAAAGGCACAAAA
